# Inflammatory response mediates cross-talk with immune function and reveals clinical features in acute myeloid leukemia

**DOI:** 10.1042/BSR20220647

**Published:** 2022-05-10

**Authors:** Fang-Min Zhong, Fang-Yi Yao, Jing Liu, Hai-Bin Zhang, Mei-Yong Li, Jun-Yao Jiang, Yan-Mei Xu, Wei-Ming Yang, Shu-Qi Li, Jing Zhang, Ying Cheng, Shuai Xu, Bo Huang, Xiao-Zhong Wang

**Affiliations:** 1 Jiangxi Province Key Laboratory of Laboratory Medicine, Jiangxi Provincial Clinical Research Center for Laboratory Medicine, Department of Clinical Laboratory, The Second Affiliated Hospital of Nanchang University, No. 1 Minde Road, Nanchang 330006, Jiangxi Province, China; 2 School of Public Health, Nanchang University, No. 461 BaYi Boulevard, Nanchang 330006, Jiangxi Province, China

**Keywords:** acute myeloid leukemia, immune function, inflammatory response, prognosis, tumor microenvironment

## Abstract

Accumulated genetic mutations are an important cause for the development of acute myeloid leukemia (AML), but abnormal changes in the inflammatory microenvironment also have regulatory effects on AML. Exploring the relationship between inflammatory response and pathological features of AML has implications for clinical diagnosis, treatment and prognosis evaluation. We analyzed the expression variation landscape of inflammatory response-related genes (IRRGs) and calculated an inflammatory response score for each sample using the gene set variation analysis (GSVA) algorithm. The differences in clinical- and immune-related characteristics between high- and low-inflammatory response groups were further analyzed. We found that most IRRGs were highly expressed in AML samples, and patients with high inflammatory response had poor prognosis and were accompanied with highly activated chemokine-, cytokine- and adhesion molecule-related signaling pathways, higher infiltration ratios of monocytes, neutrophils and M2 macrophages, high activity of type I/II interferon (IFN) response, and higher expression of immune checkpoints. We also used the Genomics of Drug Sensitivity in Cancer (GDSC) database to predict the sensitivity of AML samples with different inflammatory responses to common drugs, and found that AML samples with low inflammatory response were more sensitive to cytarabine, doxorubicin and midostaurin. SubMap algorithm also demonstrated that high-inflammatory response patients are more suitable for anti-PD-1 immunotherapy. Finally, we constructed a prognostic risk score model to predict the overall survival (OS) of AML patients. Patients with higher risk score had significantly shorter OS, which was confirmed in two validation cohorts. The analysis of inflammatory response patterns can help us better understand the differences in tumor microenvironment (TME) of AML patients, and guide clinical medication and prognosis prediction.

## Introduction

Acute myeloid leukemia (AML), as a hematological tumor, is induced by oncogenic factors affecting hematopoietic stem/progenitor cells, and the pathogenesis is still unclear [1]. Intensive treatment of AML is mainly based on combined induction chemotherapy with cytarabine and anthracyclines, but the adaptability to different populations is poor, especially for elderly patients [2]. Complex somatic mutations are closely associated with AML, and patients with different mutational signatures are highly heterogeneous. At present, a variety of targeted drugs have been developed for patients with different gene mutations, including inhibitors targeting FMS-like tyrosine kinase 3 (FLT3) and isocitrate dehydrogenase 1 and 2 (IDH1 and IDH2) [2]. For patients with corresponding mutational signatures, the use of targeted drugs has significantly improved survival and brought more hope for the treatment of AML; however, primary and secondary drug resistance is still a serious problem, which brings great challenges to clinical treatment [3]. Therefore, it is of great significance to explore more drug targets for AML treatment and basic research.

AML often disrupts the hematopoietic function of the blood system, and the perspective of overall tumor microenvironment (TME) disturbance may advance our understanding of the occurrence and development of AML. A number of studies have shown that abnormal activation of inflammatory signals favors the survival of AML cells [4,5]. By secreting a large number of pro-inflammatory cytokines such as S100A8/S100A9, interleukin (IL) 1β (IL-1β), IL-6, and IL-8, it not only promotes the growth and proliferation of AML cells, but also induces chronic inflammation to create an immunosuppressive environment for AML cells [6]. A study co-cultured primary AML cells with different cytokines and found that cytokines such as IL-1α, granulocyte-macrophage colony-stimulating factor (GM-CSF), IL-3, and tumor necrosis factor α (TNF-α) significantly promoted the growth of AML cells, and the role of IL-1β is the most obvious. They further treated 60 primary AML samples with IL-1β and found a nearly 15-fold increase in AML cell growth and survival [5]. In another study, the authors examined the levels of several pro- and anti-inflammatory cytokines in the plasma of AML patients and healthy individuals, and compared with age-matched controls, the content of TNF-α, IL-6, and IL-10 were higher in plasma of AML patients [7]. These findings all confirmed the abnormal expression of cytokines and the promoting effect of the inflammatory response they mediate on AML. Therefore, in the context of malignant hematopoiesis, it is critical to unravel the relationship of inflammatory cytokine interactions. Their mediated functions span the entire immune system, and their aberrant expression creates the conditions for a favorable TME in AML.

With the development of sequencing technology, the study of AML genomics, genetic diversity, and molecular interactions has made great progress. Currently, studies on inflammatory mechanisms in AML are mostly limited to a single molecule or a single pathway, lacking a systematic assessment of inflammatory response gene sets. Therefore, an in-depth exploration of the interaction and overall expression of these molecules can advance our understanding of the relationship between inflammatory response and the pathological features of AML.

This project systematically assessed the expression variation landscape of inflammatory response-related genes (IRRGs) in AML samples, analyzed the pathway activities and immune effects of AML cells with different inflammatory response patterns, and constructed a prognostic risk-score model. AML samples with high inflammatory response show immunosuppression, high expression of immune checkpoints and high infiltration of inflammatory cells, which are more suitable for immunotherapy; samples with low inflammatory response are more sensitive to the AML chemotherapy drugs cytarabine, doxorubicin, and the FLT3-targeting inhibitor midostaurin. Risk-score model can also accurately predict the prognosis of patients. These findings may provide new clues for exploring the inflammatory response mechanism and clinical treatment of AML.

## Materials and methods

### Data processing

**Figure 1:**
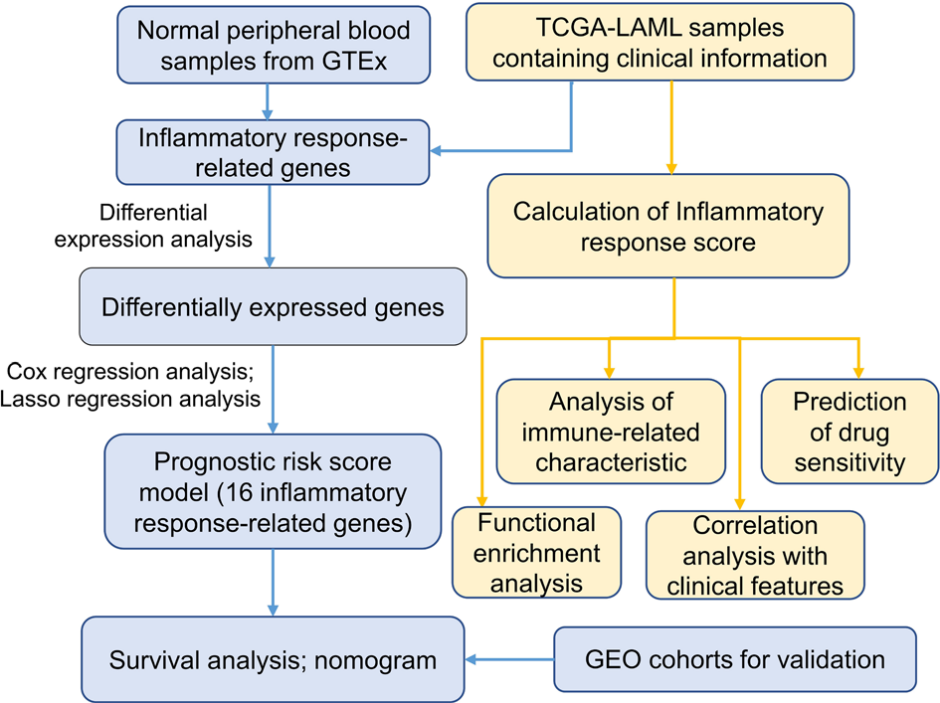
The workflow of the present study

We downloaded the RNA-sequencing data for transcripts per kilobase million (TPM) value including 173 The Cancer Genome Atlas (TCGA)-Acute Myeloid Leukemia samples and 337 normal Genome Tissue Expression (GTEx)-whole blood samples from the University of California Santa Cruz’s XENA database (https://xenabrowser.net/datapages/), these data served as the analysis cohorts. Then we downloaded two-chip data (GSE10358 and GSE71014) from the Gene Expression Omnibus (GEO) database (https://www.ncbi.nlm.nih.gov/geo/) as the validation cohorts. For the GSE10358 cohort, we downloaded the original ‘cel’ file of 91 AML samples containing clinical information, and used the function robust multiarray averaging (RMA) in the R package ‘Affy’ to standardize them. For the GSE71014 cohort, we directly downloaded the normalized data containing 104 AML samples. Somatic mutation data were downloaded from the TCGA database (https://portal.gdc.cancer.gov/). The gene set ‘HALLMARK_INFLAMMATORY_RESPONSE’ containing 200 IRRGs was downloaded from the MSigDB database (https://www.gsea-msigdb.org/gsea/msigdb/). The workflow of the present study was shown in (Figure 1).

### Identification of the differentially expressed genes between high- and low-inflammatory response groups

We used Empirical Bayesian methods via the ‘LIMMA’ package to analyze the differentially expressed genes (DEGs) between high- and low-inflammatory response groups. Genes with adjusted *P*-values <0.05 and logFC > 1 were identified as DEGs.

### Gene set variation analysis

Gene set variation analysis (GSVA) calculates the enrichment score of a gene set in a single sample based on the overall expression level of all genes in the gene set [8], and is used to quantify the activity of the corresponding biological process or signaling pathway.

### Function analysis and protein–protein interaction network

We used the R package ‘clusterProfiler’ to perform function analysis. For the signaling pathways that differ between high- and low-inflammatory response groups, we used gene set enrichment analysis (GSEA) to identify them; for the DEGs between high- and low- inflammatory response groups, we used Kyoto Encyclopedia of Genes and Genomes (KEGG) enrichment analysis and Gene Ontology (GO) annotation to analyze their function [9]. The IRRGs and the DEGs between high- and low-inflammatory response groups were uploaded to the STRING database (https://string-db.org/) for protein–protein interaction (PPI) network analysis, respectively, and Cytoscape software was used to identify core genes in PPI network.

### Evaluation of immune cell infiltration in AML samples

We used CIBERSORT algorithm to evaluate the infiltration ratio of 22 immune cells in AML samples based on the LM22 gene signatures [10] (Supplementary Table S1).

### Tumor immune dysfunction and exclusion and immune response-related gene sets

We used the tumor immune dysfunction and exclusion (TIDE) website (http://tide.dfci.harvard.edu/) to predict the TIDE score of AML samples in TCGA cohort [11]; a high TIDE score represents a stronger immune escape ability of AML cells. The immune response-related gene sets were collected for evaluating immune function of AML cell from the previous study [12], such as type I/II interferon (IFN) response, antigen-presenting cell (APC) co-inhibition/co-stimulation.

### Drug sensitivity prediction

The Genomics of Drug Sensitivity in Cancer (GDSC; https://www.cancerrxgene.org/) database was used to predict the sensitivity of each AML sample to common chemotherapy or targeted drugs [13], and the ‘pRRophetic’ package was used to calculate the half-maximal inhibitory concentration (IC_50_) value of each drug [15]. SubMap (https://cloud.genepattern.org/gp) algorithms were used to predict response to anti-PD-1 and anti-CTLA4 immune check point inhibitors in low- and high-inflammatory response groups.

### Construction of risk-score model

We first performed univariate Cox regression analysis on IRRGs with differential expression between AML samples and normal samples, genes with *P*-values <0.05 for the construction of risk-score model. Then, we used least absolute shrinkage and selection operator (LASSO) regression analysis to remove the redundant gene to avoid overfitting of the risk-score model. The penalty parameters (λ) were determined by ten-fold cross-validation. We calculated the risk score of each sample by the following formula:

$$Risk\;score=\sum_{1}^{i}\;\left(Coefi\times ExpGenei\right),$$

‘Coef’ represents the non-0 regression coefficient of each model gene and ‘ExpGene’ is the expression value of the corresponding gene (Supplementary Table S2).

### Development of a nomogram for predicting overall survival

We used R package ‘rms’ to develop a nomogram with age, cytogenetic risk and risk score based on TCGA cohort for OS prediction in AML. Then, we plotted time-dependent calibration curves to predict the accuracy of this nomogram.

### Statistical analysis

The Wilcoxon rank-sum test and the Kruskal–Wallis test were used to determine the difference between two groups and multiple groups, respectively. The ‘survminer’ package divided patients into two groups based on cutoff point. The log-rank test was used to determine *P*-values between groups in the Kaplan–Meier survival analysis. Univariate and multivariate Cox regression analyses were used to identify prognostic factors. Receiver operating characteristic (ROC) curve analysis was used to determine the specificity and sensitivity of related metrics, and the ‘pROC’ package shows the area under the ROC curve (AUC). The ‘maftools’ package was used to characterize somatic mutations of AML patients. A two-sided *P*-value of <0.05 was considered statistically significant.

## Results

### The expression variation landscape of IRRGs

**Figure 2:**
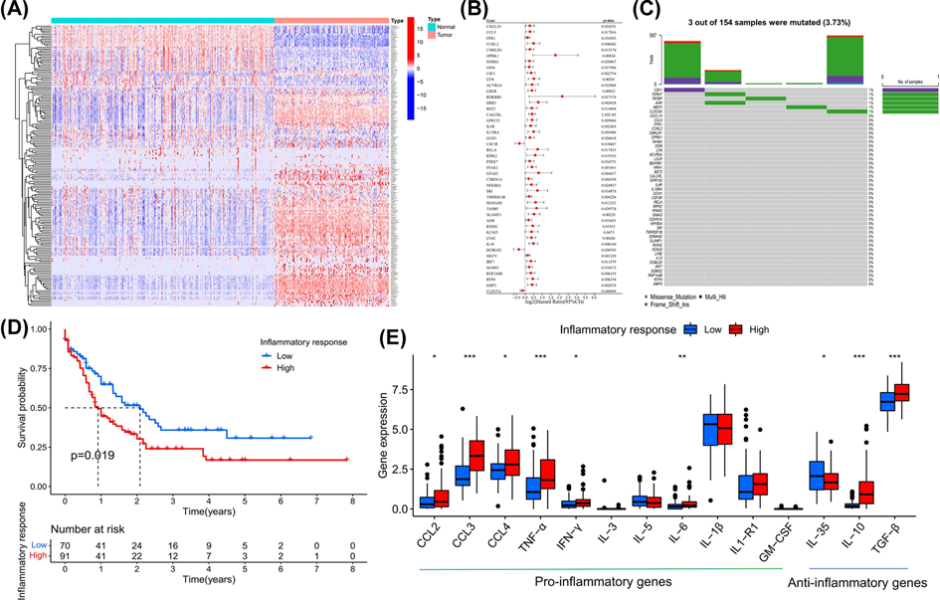
The expression variation landscape of IRRGs and prognostic analysis of inflammatory response score (**A**) Differential expression analysis of IRRGs between AML samples and normal samples, Wilcoxon test, *P<0.05; **P<0.01; ***P<0.001. (**B**) Prognosis-associated DEGs in IRRGs, Cox regression analysis. (**C**) The mutation characteristic of IRRGs in 134 AML patients from TCGA cohort. (**D**) Differences in the overall survival (OS) of patients in the high- and low-inflammatory response-score groups, log-rank test. (**E**) Differential expression analysis of pro-and anti-inflammatory genes between low- and high-inflammatory response groups.

We first analyzed the expression levels of IRRGs in the analysis cohorts; compared with the GTEx cohort, a total of 172 IRRGs with *P*<0.05 were differentially expressed in the TCGA cohort (Figure 2A), of which 54 were down-regulated and 118 were up-regulated (Supplementary Table S3). The results of functional analysis showed that the biological processes of these DEGs were mainly enriched in cytokine-mediated signaling pathways, and their molecular functions were mainly cytokine activity (Supplementary Figure S1A,B). Cox regression analysis showed that 46 DEGs with *P*<0.05 were significantly associated with the prognosis of AML patients (Figure 2B). We performed somatic mutation analysis on all IRRGs, and the overall mutation rate in AML samples was low, with only 5 of the 134 samples mutated in genes including *CLEC5A*, *MEFV*, *AHR*, *TAPBP*, *P2RX7* and *CSF1* (Figure 2C). Then we calculated the enrichment score of the inflammatory response gene set by the GSVA algorithm, and used it to represent the degree of inflammatory response in AML cell of each sample (Supplementary Table S4). Based on cutoff value, AML patients were divided into high- and low-inflammatory response groups. Survival analysis showed that patients with higher inflammatory response had significantly worse prognosis (Figure 2D).We collected pro- and anti-inflammatory genes associated with AML in a previous review [18]. Differential expression analysis showed that compared with the low-inflammatory response group, the high-inflammatory response group had higher expression levels of most pro-inflammatory genes such as CCL2, CCL3, CCL4, TNF-α, IFN-γ, IL-6 and anti-inflammatory genes such as IL-10, TGF-β, indicating an active inflammatory response of AML cells (Figure 2E).

### Differences in biological processes between patients with high- and low-inflammatory responses

**Figure 3:**
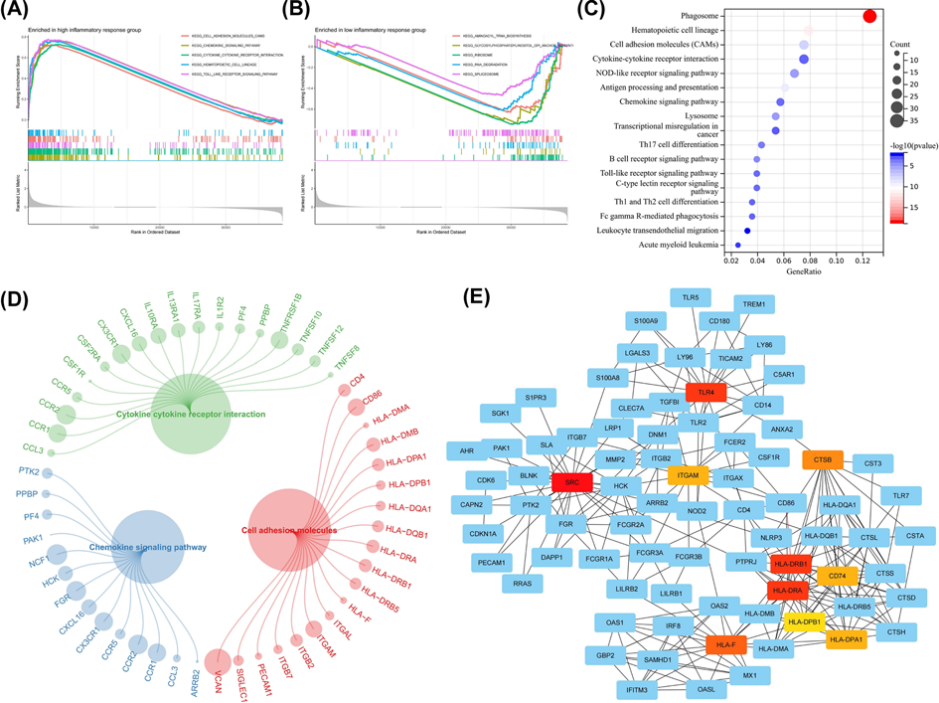
Changes in signaling pathways and identification of core molecules in high- and low-inflammatory response groups (**A**,**B**) GSEA revealed the top five signaling pathways that were significantly enriched differently between high- and low-inflammatory response groups. (A) High-inflammatory response group and (B) low-inflammatory response group. (**C**) KEGG pathway enrichment analysis of DEGs between high- and low-inflammatory response groups. (**D**) Significantly enriched signaling pathways and corresponding DEGs identified between high- and low-inflammatory response groups. (**E**) Identification of the core genes with the highest connectivity in the PPI network, the darker the color, the higher the connectivity.

To better identify the differences of biological processes in AML cell with different inflammatory responses, we used the GSEA algorithm to calculate the activity of signaling pathways in high- and low-inflammatory response groups. We observed that the top five enriched terms in the high-inflammatory response group were cell adhesion molecules, chemokine signaling pathways, cytokine–cytokine receptor interactions, hematopoietic cell lines, and TOLL-like receptor signaling pathways, all of which were closely related to inflammation (Figure 3A). In the low-inflammatory response group, signaling pathways such as aminoacyl tRNA biosynthesis, glycosylphosphatidylinositol GPI anchor biosynthesis, ribosome, RNA degradation and spliceosome have the highest enrichment scores (Figure 3B), which are all involved in the process of transcription and translation, indicating that low-inflammatory response is accompanied by more active expression of genetic information. In order to better explore the key molecules mediating high inflammatory response, we performed differential analysis between high- and low-inflammatory response groups and identified a total of 545 DEGs (Supplementary Table S5). KEGG analysis showed that these genes were also enriched in cytokine receptors-, adhesion molecules-, and chemokine-related signaling pathways (Figure 3C). Figure 3D showed the DEGs of the corresponding pathways. It is worth noting that these genes are highly expressed in the high-inflammatory response group. The PPI network displayed the top ten most connected genes in DEGs, including *TLR4*, *SRC*, *ITGAM*, *CTSB*, *CD74* and five HLA-II genes (Figure 3E).

### Immune-related characteristics of low- and high-inflammatory response groups

**Figure 4:**
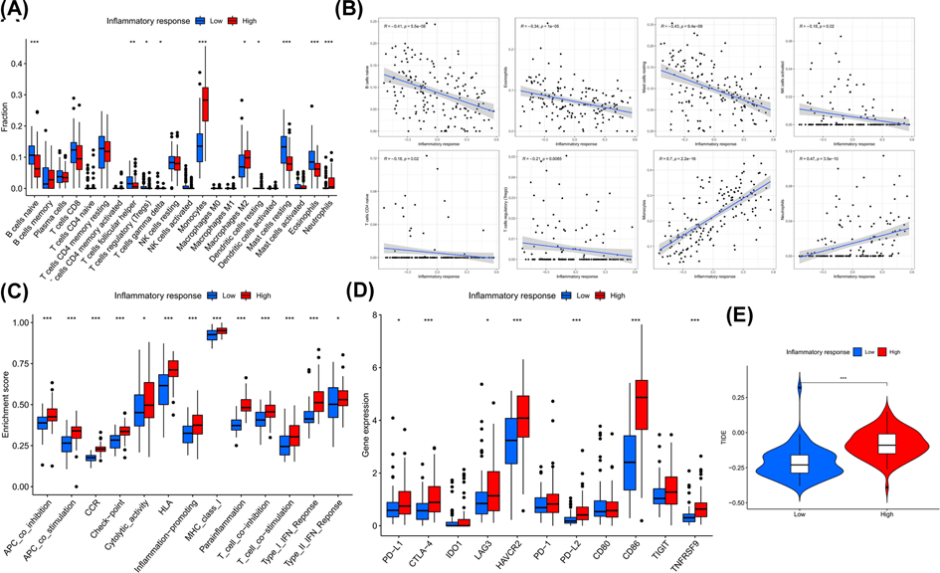
Differential analysis of immune-related characteristics between low- and high-inflammatory response groups (**A**) Infiltration levels of 22 immune cells. (**B**) Immune cells significantly correlated with inflammatory response score. (**C**) Activity of features relevant to AML cellular immune function. (**D**) Expression levels of immune checkpoints. (**E**) TIDE score.

The infiltration ratios of monocytes, M2 macrophages and neutrophils were higher in the high-inflammatory response group; naive B cells, follicular helper T cells, regulatory T cells, resting cells were abundantly enriched in the low-inflammatory response group (Figure 4A). As the inflammatory response increased, there was more infiltration of naive B cells, eosinophils, resting mast cells, activated NK cells, naive CD4 T cells, regulatory T cells, and monocytes and neutrophils; monocytes and neutrophils were less infiltrated (Figure 4B). Regarding the expression characteristics of immune function-related molecules (Supplementary Table S6), we observed that the high-inflammatory response group not only highly expressed molecules of inflammation-promotion and para-inflammation, but also CC motif chemokine receptor (CCR), human leukocyte antigen (HLA), immune checkpoints, and cytolysis- and type I/II IFN response-related molecules; co-inhibitory and co-stimulatory molecules of APC and T cells were also significantly expressed (Figure 4C). Then we compared the expression levels of common immune checkpoints between the two groups, The expressions of *PD-L1*, *CTLA-4*, *LAG3*, *HAVCR2*, *PD-L2*, *CD86*, *TNFRSF90* were significantly up-regulated in the high-inflammatory response group (Figure 4D). In addition, the high-inflammatory response group had higher TIDE score (Figure 4E). These immune signatures suggest that patients in the high-inflammatory response group are prone to immune escape.

### Clinical features and drug sensitivity of low- and high-inflammatory response groups

**Figure 5:**
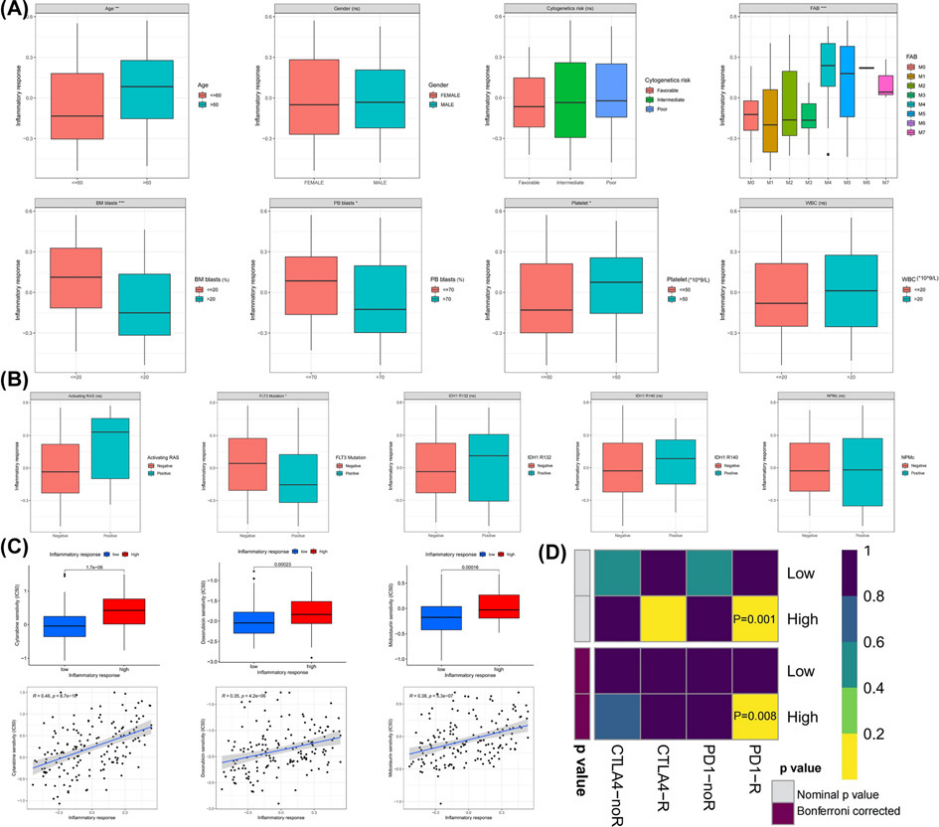
Differences in inflammatory response of AML cells in patients with different clinicopathological characteristics and prediction of sensitivity to commonly used chemotherapeutic drugs and immunotherapy response for AML (**A**) Clinicopathological features, (**B**) somatic mutation signatures, (**C**) therapeutic sensitivity of three commonly used chemotherapeutic drugs for AML. (**D**) Response prediction to immunotherapy (anti-PD-1 and anti-CTLA4) between the low- and high-inflammatory groups.

For some clinicopathological features (Figure 5A), we observed a higher inflammatory response in elderly patients (≥60 years old), which was also accompanied by counts of higher platelet, bone marrow (PB) and peripheral blood (PB) blasts. In French–American–British (FAB) classifications, M4–M7 patients had a higher inflammatory response. Besides, there were no differences between the two groups in gender, cytogenetic risk, and white blood cell (WBC) counts. For some somatic mutation signatures (Figure 5B), such as nucleophosmin cytoplasmic (NPMc), Ras activating, IDH1 mutation, and NPMc, there was no difference in the inflammatory response between positive and negative patients; but FLT3 mutation-positive patients had significantly lower inflammatory responses. We further predicted the sensitivity of patients in high- and low-inflammatory response groups to commonly used therapeutic drugs for AML based on gene expression profiles. By calculating the IC_50_ of the drug response, we found that the IC_50_ of the chemotherapeutic drugs cytarabine, doxorubicin and midostaurin, an inhibitor targeting FLT3 mutations, were lower in the low-inflammatory response group than in the high-inflammatory response group (Figure 5C), indicating that the patients with low inflammatory response are more sensitive to these therapeutic drugs, and it verified the result that patients with positive FLT3 mutation had lower inflammatory response. The high-inflammatory group exhibited features of immune escape, and we sought to explore whether these patients responded to immunotherapy. We subset-mapped the expression data profiles of AML patients with another dataset containing [47] melanoma patients who responded to immunotherapy [19]. Surprisingly, patients in the high inflammatory response group were more likely to respond to anti-PD-1 therapy (Figure 5D). (Bonferroni corrected P = 0.008).

### Construction and validation of prognostic risk-score model

**Figure 6:**
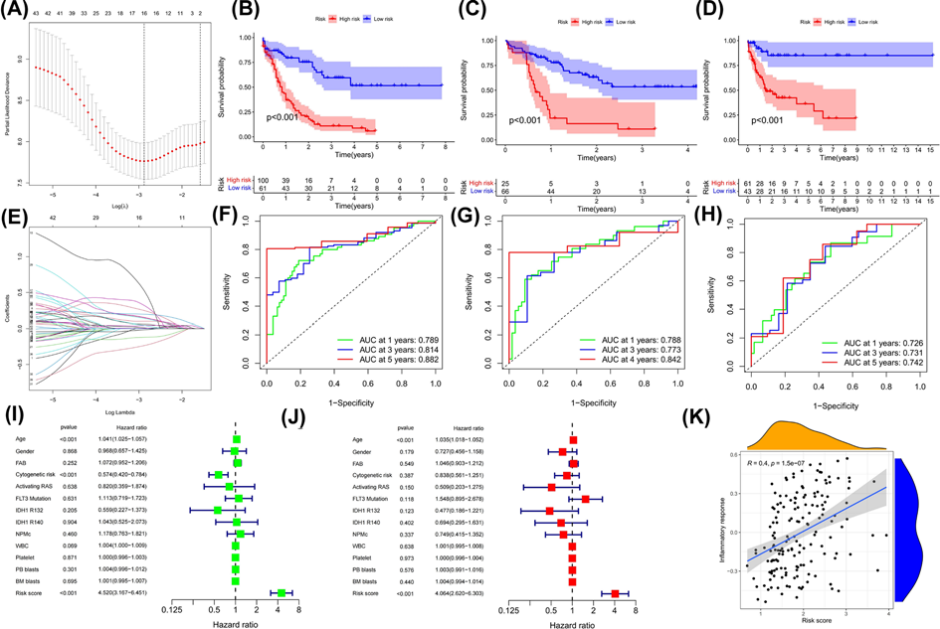
Construction and validation of the risk score model (**A**) Calculation for log(λ) of minimum ten-fold cross-validation error points and determination of corresponding model genes. (**B**) Coefficients of model genes. (**C–E**) Survival analysis between the high- and low-risk score groups in TCGA cohort and two GEO cohorts. (D) GSE10358; (E) GSE71014. Log-rank test. (**F–H**) Time-dependent ROC curve analysis of the risk score in the TCGA cohort and the GEO cohorts. (F) TCGA cohort; (G) GSE10358; (H) GSE71014. (**I,J**) Cox regression analysis of clinicopathologic factors and risk score in the TCGA cohort. (I) Univariate and (J) multivariate. (**K**) Spearman correlation analysis of risk score and inflammatory response score in the TCGA cohort.

To better predict the prognosis of AML patients, we constructed a risk score model. A total of 16 IRRGs were involved in model construction after dimensionality reduction in prognosis-related differentially expressed IRRGs using LASSO regression in the TCGA cohort (Figure 6A,B). After calculating the risk score of each sample by the model formula and sorting (The model gene coefficients are shown in Table S2), we divided AML patients into high- and low-risk score groups according to the cutoff value. Survival analysis showed that patients in the high-risk score group had significantly worse prognosis than those in the low-risk group (Figure 6C). In both the GEO cohorts, we observed the same prognostic characteristics (Figure 6D,E). In the TCGA cohort, time-dependent ROC curve analysis showed that the AUC values for predicting 1-, 3-, and 5-year OS were 0.789, 0.814, and 0.882, respectively (Figure 6F); in the GSE10358 cohort, the 1-, 3-, and 4-year (too few patients with OS over 5 years) AUC values were 0.788, 0.773, 0.842, respectively (Figure 6G); the AUC values in the GSE710148 cohort at 1, 3, and 5 years were 0.726, 0.731, and 0.742, respectively (Figure 6H). Both univariate and multivariate Cox analyses confirmed that risk scores had independent predictive power (*P*<0.05) (Figure 6I,J). Spearman correlation analysis showed that the risk score was highly positively correlated with the inflammatory response (Figure 6K). In short, validated by two GEO cohorts, the prognostic model we constructed had high accuracy.

### Development of a nomogram for predicting OS

**Figure 7:**
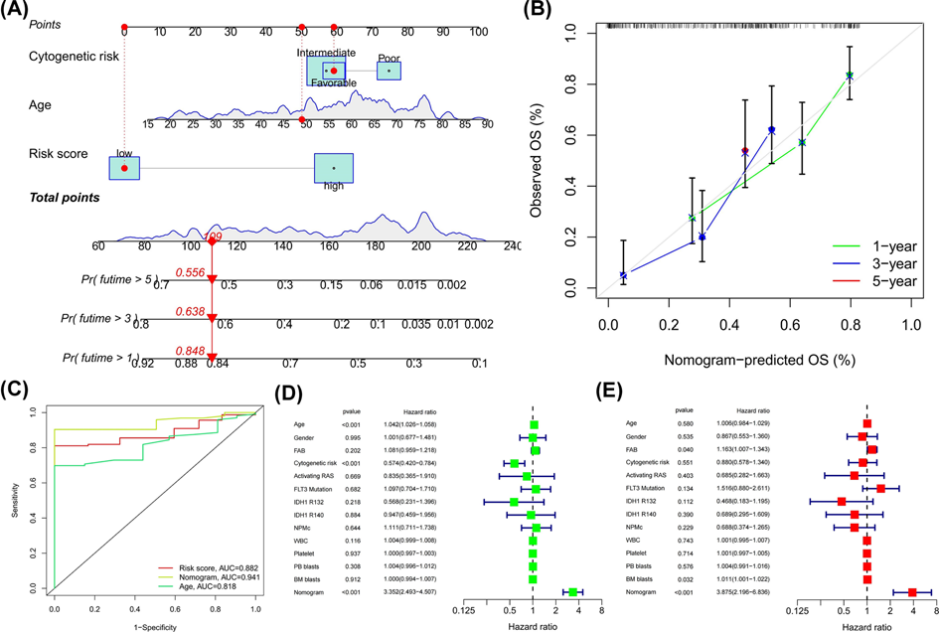
Predictive value of risk score combined with prognosis-related clinicopathological factors for overall survival (**A**) Nomogram predicting OS for AML patients in TCGA cohort. (**B**) Time-dependent calibration curves to test the predictive accuracy of the nomogram. The x-axis is nomogram-predicted OS, and the y-axis is actual OS. (**C**) ROC curves for risk score, nomogram, and age. (**D**,**E**) Cox regression analysis of the nomogram. (D) Univariate and (E) multivariate.

We observed that age and cytogenetics were significantly associated with prognosis in AML patients, so we integrated the risk score and these two clinicopathological factors to construct a nomogram to predict OS for AML patients (Figure 7A). Calibration curves at 1, 3, and 5 years demonstrated that the nomogram could predict OS accurately (Figure 7B). ROC curve analysis showed that the AUC values of risk score, nomogram, and age for predicting the 5-year OS were 0.882, 0.941 and 0.818, respectively (Figure 7C), indicating that nomogram further improved the accuracy of OS prediction. Univariate and multivariate Cox analyses also confirmed that the nomogram could serve as an independent predictor (*P*<0.05) (Figure 7D,E).

## Discussion

The survival of cancer cells is closely related to the TME in which they live. As a blood cancer, AML is affected by many physicochemical factors in the hematopoietic system [15]. In order to better survive and proliferate, AML cells secrete various cytokines to regulate the surrounding environment after being stimulated by danger [16]. The dysregulated expression of cytokines will create chronic inflammation and promote the development of AML [17]. Abnormal cytokine signaling pathways are characteristic of all types of leukemia, especially IL-1, TNF-α, and IL-6, which are considered key cytokines that disrupt hematopoietic cell function and promote the development of inflammation [18–20]. In this study, we analyzed the activity of inflammatory response gene sets in AML samples, trying to reveal the relationship between different inflammatory response signals and the immune microenvironment characteristics and clinicopathological factors of AML patients, so as to provide guidance for clinical treatment and prognosis prediction.

In this project, we found that the expression of most inflammatory response genes was up-regulated in AML samples, and these genes were mainly involved in the regulation of cytokine- and chemokine-related pathways. In the high-inflammatory response group, we observed that pro-inflammatory genes such as CCL2, CCL3, CCL4, TNF-α, IFN-γ, andIL-6are generally highly expressed, and many studies have confirmed that they promote the survival and reproduction of AML cells [7, 26–28]; we also noticed that classical anti-inflammatory cytokines such as IL-10 and TGF-β showed high expression levels, which tend to inhibit the proliferation of AML cells, but these studies were for exogenous cytokines such as in plasma. Autocrine or paracrine TGF-β by leukemia cells is able to exert negative control over the growth of normal progenitor cells, but not leukemia cells, which have overcome TGF-β regulatory signals [29]. Similarly, exogenous IL-10 is thought to have inhibitory effects on AML cells [30], but the study of endogenous IL-10 is rarely reported, which requires further research. In conclusion, the high-inflammatory response group showed a positive pro-inflammatory effect, and these patients also corresponded to a poor prognosis, indicating that elevated inflammatory responses in AML cells are closely related to AML development. In addition to cytokines and chemokines, the DEGs identified by the high- and low-inflammatory response groups were up-regulated in the expression of a large number of adhesion molecules. The high expression of these molecules may promote AML cells to adhere to the bone marrow niche [21], thereby evading the effect of drugs or the attack of immune cells. We identified several core molecules in DEGs, such as TLR4, SRC, ITGAM and CTSB, through the PPI network. Among them, the expression of TLR4 is associated with poor prognosis and can be inhibited with chemosensitizing and/or direct anti-leukemia effects [22–24]; knockdown of CTSB can inhibit the proliferation and tumorigenesis of the AML cell line HL-60 [25], which is also a poor prognostic factor for childhood AML [26]; SRC is a signaling mediator that activates STAT5 in FLT3-ITD-positive AML patients [27], and its overexpression also affects the sensitivity of FLT3-ITD kinase inhibitor and the pathway of acquired resistance [28]; ITGAM is a risk factor for autoimmune diseases such as systemic lupus erythematosus [29], but the mechanism associated with AML is less studied. Therefore, these molecules have potential as therapeutic targets for AML, but further exploration is required.

Inflammatory response interacts with immune function, and we further explored the differences in immune-related signatures in different inflammatory-response samples. The high-inflammatory response group had more inflammatory cell infiltration, including monocytes, neutrophils, and M2 macrophages, which produce immunosuppression during tumor development by releasing cytokines to induce chronic inflammation, and also enhance the secretion of inflammatory factors in tumor cells to further deteriorate the tumor micro environment [40–43], while immune killer cells such as T cells, B cells, and NK cells were rarely enriched. In addition, we observed more eosinophilic infiltration in the low-inflammatory response group. Studies have shown that IL5 can induce an increase in the number of eosinophils in AML [44,45]. When we compared the differences in the expression of pro- and anti-inflammatory genes, we found that the expression level of IL-5 was higher in the low-inflammatory group compared to the high-inflammatory group. The secretion of IL-5 may be responsible for the greater infiltration of eosinophils. Other studies have reported that AML patients with M1 and M2 types are often associated with eosinophilia [46], and both subtypes are more common in the low-inflammatory response group, which may also be associated with more eosinophilic infiltration. The high-inflammatory response group also showed higher HLA expression and IFN response. High expression of HLA genes is a unique signal of blood cells, and it is no exception in AML cells [47]. Elevated IFN responses often imply a poor prognosis, possibly due to IFN-driven immune resistance [48]. The co-inhibitory molecules of T cells are mainly immune checkpoints such as PD-L1 and CTLA4 [49], which are generally highly expressed in the high-inflammatory response group, showing a strong ability to suppress T cells; while co-stimulatory molecules of T cells such as CD28 are mainly expressed on the surface of T cells, they mediate the second signal of full activation of T cells, and thus participate in the protective function of the immune system [50]. However, the reasons for their high expression in AML cells are rarely reported. We hypothesized that they would promote the recognition of tumor cells by T cells to exert anti-tumor effects, but the co-stimulatory molecule CTLA4 is also highly expressed, and CTLA4 competes with CD28 to bind ligands such as CD80 and CD86 in T cells and has higher affinity, thereby overcoming the positive effects of CD28 and placing T cells in an immunosuppressive state. This hypothesis requires more experiments and data to verify. Similarly, APC co-inhibitory molecules are mainly immune checkpoints such as PD-L1 and LGALS9 [51], which are highly expressed in AML cells to inhibit the activity of APC. CD40 is mainly involved in the co-stimulation of APCs, and its high expression is associated with poor prognosis in AML, and is mainly expressed in M4 and M5 patients [52]. CD40 can promote the proliferation of AML cells and inhibit apoptosis [53], and the IFN response can also increase its molecular expression in AML cells [54], thereby promoting the production of cytokines. Therefore, elevated activities of APC co-inhibitory and co-stimulatory molecules have the effect of inducing the development of inflammation and protecting AML cells. We also observed high TIDE score in the high-inflammatory response group, these immune-related features suggest that the activation of the inflammatory response in AML cells exhibits an inflammatory and immunosuppressive microenvironment, which in turn promotes immune escape of AML cells. In the subsequent prediction of immunotherapy response, we found that the high-inflammatory response group was likely to respond to anti-PD-1 treatment. We also predicted the sensitivity of different-inflammatory response samples to drug treatment. As the inflammatory response of AML cells increased, the chemotherapeutic drugs cytarabine and doxorubicin and the FLT3-targeting inhibitor midostaurin showed lower sensitivity. Combined with the relationship between inflammatory response and immune characteristics mentioned above, we believe that these three therapeutic drugs are more suitable for patients with low-inflammatory response of AML cells. For patients with a high inflammatory response, immunotherapy targeting immune checkpoints or inflammatory cells such as M2 macrophages is more applicable.

Some clinicopathological features also showed differences in inflammatory responses. For example, patients with M4-M7 subtypes and a small number of patients with M2 subtype show a higher inflammatory response. Studies have shown that M4 and M5 subtypes with monocyte differentiation and part of myeloid mature M2 subtype in AML produce more inflammatory chemokines [55]; while inflammatory genes such as TGF-β are highly expressed in M6 and M7 subtypes, which may be the reason for promoting high inflammatory response [29,56]. Compared with FLT3 mutation-negative patients, FLT3 mutation-positive patients had a lower inflammatory response, and FLT3-mutated AML cells were previously thought to be less responsive to exogenous cytokines, that is, exhibit lower inflammatory responses [57]. Another study showed that the FLT3 inhibitor quizartinib upregulates inflammatory genes in AML cells, and combined with anti-inflammatory glucocorticoids enhanced cell death in FLT3 mutants, but not wild-type [58]. AML patients with activated RAS mutation also exhibit high inflammatory responses, and an article by Hamarsheh et al. showed that AML patients with KRAS mutations, a subtype of the RAS gene, have activated inflammasome pathways, with NLRP3/caspase1/IL-1β being the major contributing axis [59].Therefore, these results reveal the heterogeneity of inflammatory responses in different clinicopathological features, and the combined assessment of them can be helpful for the diagnosis and treatment of AML.

Finally, we constructed a prognostic risk-score model including 16 IRRGs by LASSO regression analysis to predict OS for AML patients. Survival analysis showed that patients with higher risk scores had significantly poor prognosis, and ROC curve analysis confirmed the predictive accuracy of the model. We performed validation on two GEO cohorts, both showing high predictive performance. In addition, we combined the risk-score model with some prognosis-related clinical indicators to draw a nomogram, which further improved the accuracy and intuitiveness of the model for OS prediction.

In conclusion, we comprehensively analyzed the expression variation landscape of inflammatory response-related molecules in AML samples and revealed differences in immune-related signatures across high- and low-inflammatory response score groups. Inflammatory response score can also predict sensitivity to commonly used drugs for AML. Moreover, the risk score model we constructed accurately predicted OS of patients. These findings provide a new reference for clinical treatment, a new method for prognosis prediction, and enlightenment for more basic experiments related to inflammatory response.

## Supplementary Material

Supplementary Figure S1

Supplementary Tables S1-S6

## Data Availability

The RNA-seq data (TPM) from Illumina HiSeq RNASeq platform consisted of 173 LAML samples and 337 normal GTEx-whole blood samples were acquired from UCSC XENA database (https://xenabrowser.net/datapages/). In addition, gene expression profiles of datasets GSE10358 (https://www.ncbi.nlm.nih.gov/geo/query/acc.cgi?acc=GSE10358) and GSE71014 (https://www.ncbi.nlm.nih.gov/geo/query/acc.cgi?acc=GSE71014) were obtained from GEO database.

## Abbreviations

AML: acute myeloid leukemia
APC: antigen-presenting cell
AUC: area under the ROC curve
DEG: differentially expressed gene
FLT3: FMS-like tyrosine kinase 3
GEO: Gene Expression Omnibus
GSEA: gene set enrichment analysis
GSVA: gene set variation analysis
GTEx: Genome Tissue Expression
HLA: human leukocyte antigen
IC_50_: half-maximal inhibitory concentration
IDH: isocitrate dehydrogenase
IFN: interferon
IL: interleukin
IRRG: inflammatory response-related gene
KEGG: Kyoto Encyclopedia of Genes and Genomes
LASSO: least absolute shrinkage and selection operator
OS: overall survival
PPI: protein–protein interaction
ROC: receiver operating characteristic
TCGA: The Cancer Genome Atlas
TIDE: tumor immune dysfunction and exclusion
TME: tumor microenvironment
TNF-α: tumor necrosis factor α
