# Supplementary material for: Inflammatory response mediates cross-talk with immune function and reveals clinical features in acute myeloid leukemia
Source: Biosci Rep. 2022 May 10;42(5):BSR20220647. doi: 10.1042/BSR20220647 (PMC9093697; doi:10.1042/BSR20220647)
Supplement: Supplementary Figure S1 [file BSR-2022-0647_supp.pdf]

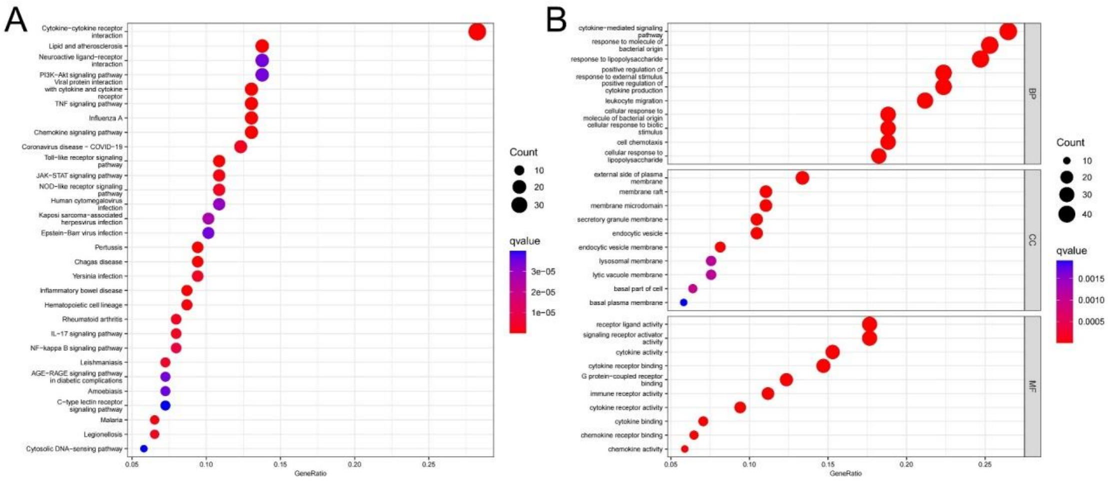

Figure S1. Functional analysis of differentially expressed inflammatory response-related genes identified between AML samples and normal samples. (A) Kyoto Encyclopedia of Genes and Genomes pathway enrichment analysis. (D) Gene Ontology annotations.
